# Three Immunocompetent Small Animal Models That Do Not Support Zika Virus Infection

**DOI:** 10.3390/pathogens10080971

**Published:** 2021-07-30

**Authors:** Megan R. Miller, Anna C. Fagre, Taylor C. Clarkson, Erin D. Markle, Brian D. Foy

**Affiliations:** Center for Vector-Borne Infectious Diseases, Department of Microbiology, Immunology and Pathology, Colorado State University, Fort Collins, CO 80523, USA; anna.fagre@colostate.edu (A.C.F.); taylorcclarkson@hotmail.com (T.C.C.); erin.markle@ucdconnect.ie (E.D.M.); brian.foy@colostate.edu (B.D.F.)

**Keywords:** ZIKV, animal models, flavivirus

## Abstract

Zika virus (ZIKV) is a mosquito-borne flavivirus that is primarily transmitted to humans through the bite of an infected mosquito. ZIKV causes disease in infected humans with added complications of Guillain-Barré syndrome and birth defects in infants born to mothers infected during pregnancy. There are several large immunocompetent animal models for ZIKV including non-human primates (NHPs). NHP models closely reflect human infection; however, due to sample size restrictions, investigations into the effects of transmission route and the impacts on disease dynamics have been understudied. Mice have been widely used for modeling ZIKV infection, yet there are few ZIKV-susceptible immunocompetent mouse models and none of these have been used to investigate sexual transmission. In an effort to identify a small immunocompetent animal model to characterize sexual transmission of ZIKV, we attempt experimental infection of multimammate mice, New Zealand white rabbits, and Hartley guinea pigs. The multimammate mouse is the natural reservoir of Lassa fever virus and has been identified to harbor other human pathogens. Likewise, while NZW rabbits are susceptible to West Nile virus, they have not yet been examined for their susceptibility to infection with ZIKV. Guinea pigs have been successfully used as models for ZIKV infection, but only in immunocompromised life stages (young or pregnant). Here, it was found that the multimammate mouse and New Zealand White (NZW) rabbits are not susceptible ZIKV infection as determined by a lack viral RNA in tissues and fluids collected. Sexually mature male Hartley guinea pigs were inoculated subcutaneously and by mosquito bite, but found to be refractory to ZIKV infection, contrary to findings of other studies in young and pregnant guinea pigs. Interestingly, here it is shown that adult male guinea pigs are not susceptible to ZIKV infection, even when infected by natural route (e.g., mosquito bite). Although a new small animal model for the sexual transmission for ZIKV was not established through this study, these findings provide information on outbred animal species that are not permissive to infection (NZW rabbits and multimammate mice) and new information surrounding limitations of a previously established animal model (guinea pigs).

## 1. Introduction

ZIKV is a positive-stranded RNA virus in family *Flaviviridae*. ZIKV is primarily transmitted to humans through the bite of an infected mosquito. Transmission also occurs perinatally, through sexual activity, and blood transfusion [1,2,3,4,5]. The 2015–2016 ZIKV pandemic in the Americas resulted in over 1 million suspected cases, with hundreds of spontaneous abortions reported and thousands of infants born with microcephaly, ocular malformations and other birth defects [6,7,8]. Following the epidemic, many groups sought to characterize animal models for ZIKV infection as a means of better understanding viral pathogenesis and the species’ immune response for future pre-clinical studies.

Non-human primates (NHPs) and mice are the most widely used animal models for ZIKV infection [9,10,11,12]. There are advantages and disadvantages to both models. Mice are small, have a fast reproductive rate, and are easy to genetically manipulate. However, immunocompetent mice are not naturally susceptible to ZIKV infection [13].

There are several large immunocompetent animal models for ZIKV—including goats, sheep, water buffalos, lions, and NHPs [14]. The NHP models are the most favorable for ZIKV owing to anatomic and physiologic similarity between humans and NHPs. NHPs are naturally susceptible to ZIKV infection and are similar to humans anatomically and physiologically, including developmentally and in utero, including comparable gestational periods [12]. Challenge studies in rhesus (*Macaca mulatta*), pigtail (*Macaca nemestrina*), and cynomolgus macaques (*Macaca fascicularis*) have shown that viremia lasts for weeks even in the absence of clinical symptoms [15,16]. This holds true for other NHPs such as owl monkeys (*Aotus* sp.), squirrel monkeys (*Saimiri* sp.), and the marmoset (*Callithrix jacchus*) [17,18].

The use of NHP models has provided guidance and information on the safety and efficacy of vaccine and drug treatments. However, they are also costly to maintain, have restrictions on group size, and their use is surrounded by ethical considerations [19]. Therefore, the integration of NHPs into studies characterizing how transmission route impacts disease outcome is limited.

Mice have been extensively used for modeling ZIKV infection, as reviewed in Bradley et al. [14]. However, very few ZIKV-susceptible immunocompetent mouse models have been established since efforts began in 2015, and none of these studies investigated sexual transmission [20,21]. All non-NHP studies establishing sexual transmission of ZIKV have relied on genetically modified knockdown mice lacking fully intact IFN 1 response [22,23]. Strains of mice successfully established for investigation of vaccines and other therapeutics include *Ifngr1* knockout, *Stat2* knockout, *Irf3/Irf5* double knockout *Irf3/Irf5/Irf7* triple knockout [9,10,24,25]. ZIKV is able to evade human type I interferon (IFN) response due to species-specific evasion mechanisms [13]. However, the IFN response in mice is able to interfere with viral replication and prevent infection [26]. While these models are helpful in assessing transmission routes, a major limitation is their inability to provide information about the immune response mounted in the face of infection.

To investigate potential small animal models with intact innate immune systems for studying the sexual transmission of ZIKV, we experimentally inoculated the New Zealand white (NZW) rabbit (*Oryctolagus cuniculus*), Natal multimammate mouse *(Mastomys natalensis),* and Hartley guinea pig (*Cavia porcellus*). These three animal models were of particular interest owing to previous studies characterizing their susceptibility to ZIKV (Hartley guinea pigs [26,27,28,29,30]) or susceptibility to other flaviviruses (NZW rabbit [31] and multimammate mouse [32]). Further, these animal models are outbred, which more closely mimic human and free-ranging animal populations in their genetic heterogeneity and allow for analysis of diverse responses to vaccines and other therapeutics [33,34].

Hartley guinea pigs have been used for a model of ZIKV infection and are susceptible to ZIKV when infected subcutaneously and intranasally [27,28]. Advantages to using guinea pigs as an animal model are their small size and high reproductive rate, facilitating the use of larger sample sizes. The reproductive physiology of the guinea pig is also similar to that of humans, making them optimal for translational animal models [35]. Recent studies have examined the effects of ZIKV on fetal development when females are infected during pregnancy, showing that pregnant dams are susceptible to infection, resulting in abnormal pregnancies [30]. However, all studies with Hartley guinea pigs have investigated susceptibility of very young animals or animals that may otherwise be immunocompromised (for instance, due to pregnancy). The susceptibility of sexually mature adult guinea pigs has not yet been assessed, nor has the potential for guinea pigs to transmit ZIKV sexually.

Challenge of New Zealand White rabbits with ZIKV has not yet been reported, though they have been established as animal models for West Nile virus and Murray Valley encephalitis virus, two other mosquito-borne flaviviruses [31]. When inoculated, NZW rabbits demonstrate a refractory phenotype similar to that appreciated in horses and humans [33]. Another interesting study showed that cottontail rabbits (*Sylvivagus* spp.) inoculated with Asian-lineage ZIKV (PRVABC59) were shown to seroconvert 28 days post-infection, though none demonstrated viremia [36]. To date, the susceptibility of NZW rabbits to ZIKV has not been described.

Lastly, the multimammate mouse is known to be a host for several viruses, including arenaviruses and flaviviruses [37,38]. Usutu virus, a *Culex*-associated mosquito-borne flavivirus, was isolated from three multimammate mice in Senegal, warranting further investigation into this rodent’s role in sylvatic flavivirus transmission [33]. Additionally, the closely related *Mastomys coucha* are used for pre-clinical models in papillomavirus research [39]. To date, there are no published studies with these animals examining their potential as a viral reservoir for medical important pathogens.

ZIKV is primarily transmitted by mosquito bite. Several studies have demonstrated that mosquito transmission, as compared to needle inoculation, of West Nile Virus (WNV) [40,41], dengue viruses (DENV) [42], Semliki Forest virus (SFV) [43] and chikungunya virus (CHIKV) [44] affect infection outcome. A study in ZIKV-infected NHPs resulted in delayed viremia when the animals were infected by mosquito bite, as well as differences in tissue tropism from individuals who were subcutaneously inoculated [45]. These results suggest that inoculation by infected mosquito bite alters replication kinetics and pathogenesis, and thus, investigating the effect of mosquito saliva is an important area of study when establishing an animal model.

Despite the devastating impacts of ZIKV and its rapid global spread in 2015–2016, to date no immunocompetent small animal model exists allowing for the study of sexual transmission dynamics and associated pathology. This study was undertaken to identify potential candidates for such a model to characterize mechanisms underpinning the sexual transmission of ZIKV.

## 2. Results

### 2.1. Multimammate Mice (Mastomys natalensis) Are Not Susceptible to ZIKV

**Figure 1:**
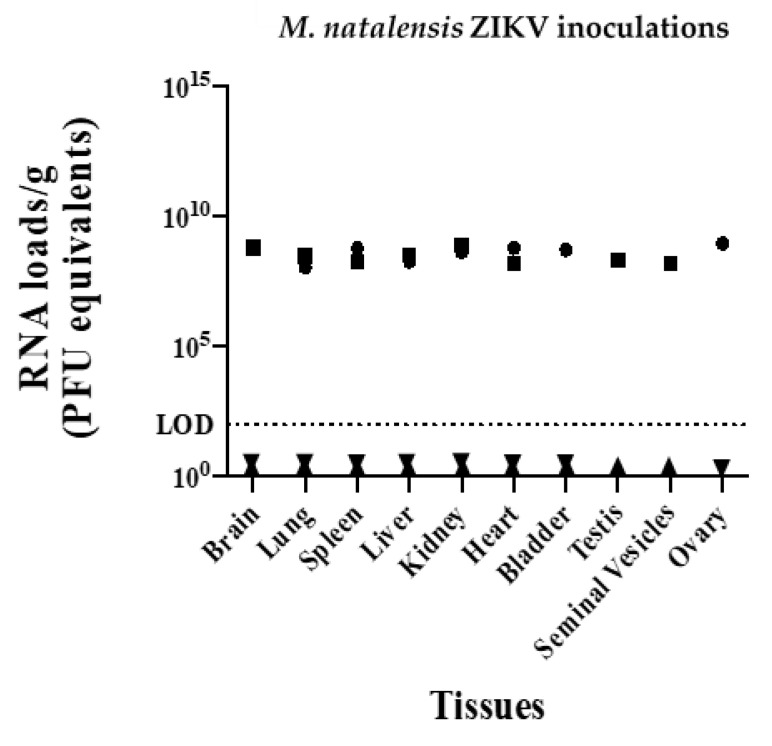
ZIKV RNA levels in inoculated *M. natalensis* samples at 5 dpi; rightside-up triangle = male, upside-down triangle = female and ZIKV RNA levels of infected *Mus musculus* (A129 strain) positive controls sampled at 5 dpi; square = male, circle = female. LOD = limit of detection.

A total of 15 multimammate mice, both male and female, were inoculated with Asian lineage ZIKV (PRVABC59) and an African strain of ZIKV (DAR41525) (Table 1 and Table 2). Additionally, two A129 mice (previously confirmed to be susceptible [9]) were infected in parallel as positive controls and were inoculated with ZIKV PRVABC59 only (Table 2). All animals were inoculated with 2.6 × 10^6^ PFU of respective ZIKV strains. All mice were euthanized five days post-inoculation, and all tissues (saliva, blood, brain, heart, lungs, liver, kidney, bladder, testis, seminal vesicles and ovary) were negative by qRT-PCR. All tissues from positive control A129 mice were positive for ZIKV viral RNA loads (10^8^–10^9^ PFU equivalents/gram) (Figure 1).

### 2.2. New Zealand White Rabbits Are Not Susceptible to ZIKV

**Figure 2:**
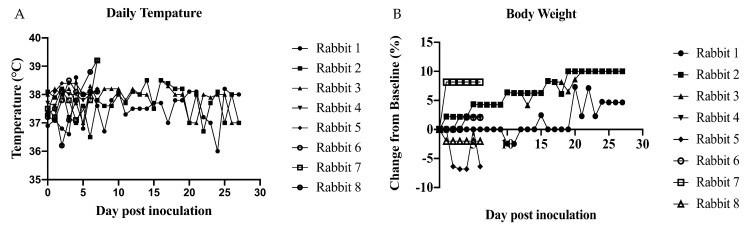
Daily measurement of inoculated rabbits taken q24 h. (**A**) Daily temperature and (**B**) body weight. No significant in changes of temperature or body weight over time. Determined by multiple *t*-test between SC and control group at each time point.

**Figure 3:**
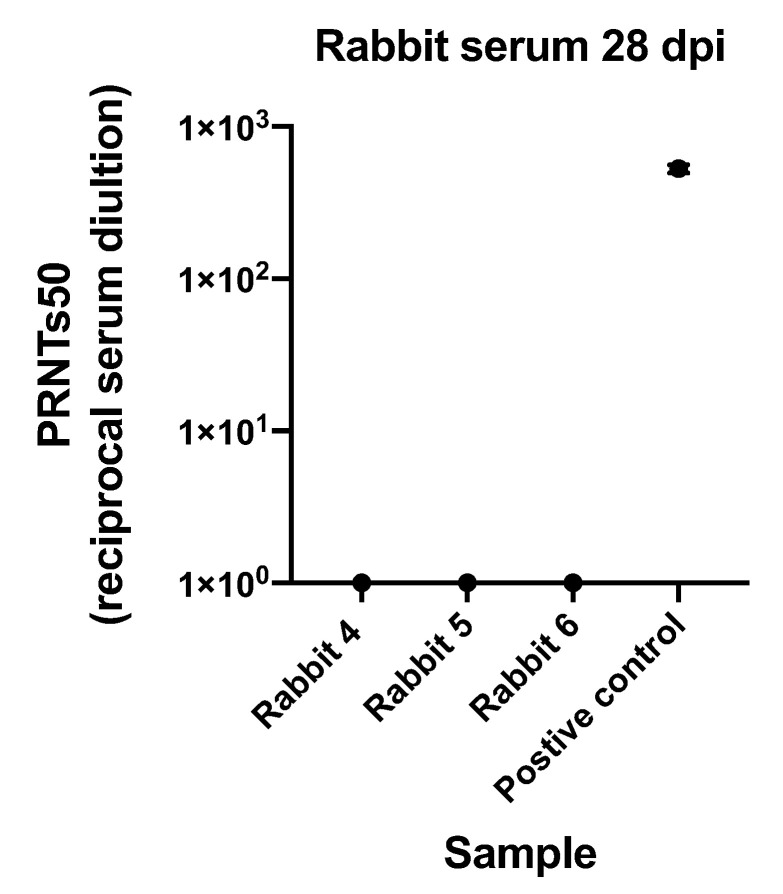
Plaque reduction neutralization test (PRNTs) on serum collected from rabbits euthanized 28 dpi compared to positive control from previously infected individual. The mean and standard deviation of three replicates are shown for each sample.

As our preliminary investigations sought to characterize male-to-female sexual transmission using the Asian lineage, the susceptibility of male rabbits to PRVABC was carried out first. Four male rabbits were subcutaneously inoculated with 2.6 × 10^6^ PFU of ZIKV PRVABC59 and two were mock-inoculated to serve as negative controls (Table 3). No significant change in temperature was observed during the course of the study between inoculated animals compared to control animals or baseline. There was no significant change in body weight between inoculated animals and control animals. However, changes in individual body weight were observed with a 10% fluctuation from baseline (Figure 2). Additionally, all tissues (brain, heart, lungs, liver, kidney, bladder, testis, and seminal vesicles) and fluid samples (saliva, blood, and semen) were negative by qRT-PCR. After male rabbits were euthanized, the two female rabbits that had been used to stimulate mating for semen collection were intravaginally inoculated with 2.6 × 10^6^ PFU of ZIKV PRVABC59 (Table 3). Blood, saliva, and vaginal swabs samples from females were negative by qRT-PCR, and females were moved into another study. Serum collected only from the 3 males euthanized at 28 dpi did not neutralize ZIKV, indicating lack of seroconversion (Figure 3).

### 2.3. Mature Hartley Are Not Susceptible to ZIKV by Mosquito Bite

**Figure 4:**
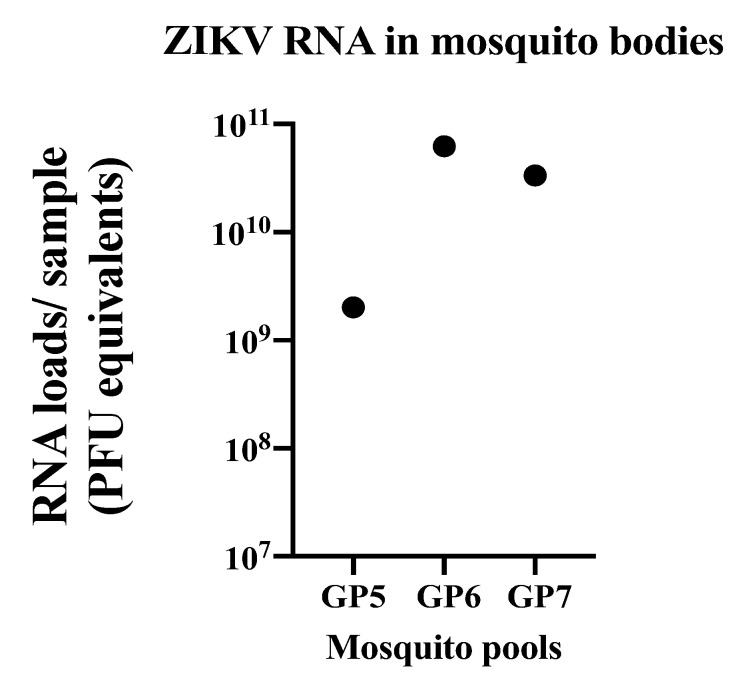
ZIKV RNA levels in pools of mosquito bodies that fed on guinea pigs (GP). Each dot represents the value of RNA in each sample.

**Figure 5:**
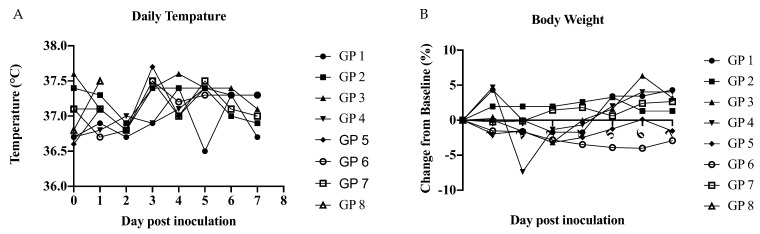
Daily measurement of inoculated guinea pigs (GP) taken q24 h. (**A**) daily temperature and (**B**) body weight. No significant in changes of temperature or body weight over time. Determined by multiple t-test between SC or MB inoculated groups compared to control at each time point.

Males were once again used to evaluate the use of these animals as a model of sexual transmission of ZIKV. Males were inoculated either subcutaneously or by infectious mosquito bite to evaluate whether mosquito saliva would potentiate infection, as multiple studies have demonstrated them to be susceptible to ZIKV infection (Table 1 and Table 4). Our previously published work has successfully infected A129 by mosquito bite, using identical methods to the current study [46]. Following infection by mosquito bite, mosquito bodies (pooled by guinea pig) were all positive for ZIKV RNA via qRT-PCR (Figure 4). However, all tissues (brain, heart, lungs, liver, kidney, spleen, bladder, and testis) and fluid samples (saliva and blood) collected from guinea pigs either infected subcutaneously or by infectious mosquito bite were negative by qRT-PCR. Daily temperature was collected q24 h and no significant changes were observed between inoculated animals and control animals or compared to baseline. Changes in body weight were observed for individual animals with a 7% fluctuation from baseline (Figure 5). Additionally, no clinical signs (fatigue, weight loss, hunched posture, scruffy fur or labored breathing) were observed (Figure 5).

## 3. Discussion

There are many murine models for the study of ZIKV, however most are immunodeficient or immunosuppressed mice that lack an intact IFN pathway. NHP models have been successfully used but the restrictions on sample size limits statistical power. Much was learned about ZIKV pathogenesis following the expansion of Asian lineage ZIKV, though many questions remain—questions whose answers may lay important groundwork for the study of other emerging viruses capable of sexual transmission. Establishment of an immunocompetent small animal model for the sexual transmission of ZIKV would help illustrate a more comprehensive portrait of ZIKV transmission and pathogenesis, informing future drug development studies and risk mitigation strategies.

We examined the multimammate mouse, New Zealand white rabbit, and Hartley guinea pig as immunocompetent small animal models for ZIKV infection with the goal of developing one into a model for studying ZIKV sexual transmission. In these studies, it was found that the multimammate mouse and NZW rabbit are not susceptible ZIKV infection. Our data also show that sexually mature Hartley guinea pigs were also not susceptible to ZIKV infection, contrary to other studies [27,28,29,30]. Additionally, ZIKV infection of guinea pigs was not established even when inoculated by infected mosquito bite. Because our preliminary goal of this study was to establish a ZIKV sexual transmission model, we focused on male infections since upwards of 90% of sexually transmitted ZIKV cases stem from male-to-female transmission [47]. Here, the susceptibility of only male animals was determined with the guinea pigs and to a lesser extent rabbits, resulting in a sex bias in these studies.

We were interested in the susceptibility of the multimammate mouse to ZIKV not only for its use as an animal model, but in characterizing potential sylvatic reservoirs of ZIKV owing to the geographic overlap of the multimammate mouse and ZIKV risk (as characterized by *suitable Aedes aegypti* habitat), in addition to the fact that mosquitoes known to transmit ZIKV (genus *Aedes*) are known to feed on rodents [48,49,50]. Although the results of our study indicate that the multimammate mouse was not susceptible to ZIKV, it plays an important role in viral ecology and its permissiveness to other viruses should be further explored in controlled settings. The multimammate mouse is natively found in West Africa and is the main reservoir of Lassa virus (LASV). The prevalence of LASV in multimammate mouse can be 8–30% in the wild, and it can be transmitted to humans by direct or indirect exposure to infected rodent fluids [48]. Other viruses have been isolated from naturally infected multimammate mouse populations, including alphaviruses, bunyaviruses and flaviviruses [49].

Previous studies inoculating Hartley guinea pigs via subcutaneous and intranasal inoculation resulted in low levels of viremia and effects on fetal development. However, guinea pigs used in these studies were either very young (under 5 weeks) [27,28,29] or pregnant [30]. One study used 6-month-old non-pregnant females, and observed low levels of RNAemia (10^3^–10^4^ RNA copies), but these animals were also subcutaneously inoculated with higher titers than we used (10^7^ or 10^8^ PFU compared to the 2.6 × 10^6^ PFU used in this study)—titers much higher than those isolated from the salivary glands of infected mosquitoes [32]. Younger animals have a less robust immune response [51] and pregnancy leads to an immunosuppressed state [52], making individuals more prone to viral infection. Therefore, it is interesting that the sexually mature guinea pigs used in our study did not become infected with ZIKV at detectable levels, and that infection by mosquito bite did not potentiate infection, as in other studies demonstrating altered replication kinetics in the presence of arthropod saliva [45]. Although guinea pigs may not be a good model for sexual transmission, they may be useful to address question surrounding fetal development during infection.

Other small animal models including hamsters, humanized STAT2 mice, and more recently the treeshrew, have been used in ZIKV infection studies [53,54]. Overall, these models, with the exception of the treeshrew, are insufficient models for sexual ZIKV transmission. Syrian golden hamsters developed neutralizing antibodies after inoculation with ZIKV, but no viremia was detected [54]. In addition, a STAT2 humanized mouse was created to make a more fully immunocompetent animal model [20]. Although this model does allow for ZIKV replication and has been used to look at drug candidates and effects on pregnancy, it is a transgenic mouse and still lacks a fully intact immune system comparable to humans. However, the treeshrew proved to be susceptible to ZIKV with high viremia and viral RNA secreted in saliva. These animals also developed typical dermatological manifestations [54]. The treeshew seems to be a promising animal model for ZIKV pathogenesis and future efforts should investigate its susceptibility via different transmission routes, including via coitus and infectious mosquito bite.

Interestingly, different ZIKV strains result in variable pathogenicity, both in clinical cases and animal models. Previous studies demonstrated that the African strain of ZIKV causes more severe infection, including in utero [55,56]. In vivo studies in which mice were infected with different strains of ZIKV also demonstrate variation in tissues tropism [57], neuropathology [58] and innate immune response [59]. Comparative studies with multiple strains of ZIKV are critical for defining genetic variation that may contribute to differences in immune response, pathology, and forward transmission potential. In this study, susceptibility of the multimammate mouse was not impacted by ZIKV strain, as all animals were refractory to all ZIKV strains used.

While our investigation did not result in the establishment of a small animal model for the sexual transmission of ZIKV as we had hoped, the findings are still valuable as we have demonstrated three outbred animal species that are not permissive to infection, one of which was confirmed susceptible at more immunocompromised life stages (the Hartley guinea pig). Immunocompetent small animal models should continue to be investigated for use in sexual transmission studies to more fully characterize at which point in infection the risk of transmission between sexual partners is highest. These data will guide future animal model work for sexually transmitted viruses.

## 4. Materials and Methods

### 4.1. Virus and Cells

African Green Monkey kidney cells (Vero; ATCC #CCL-81) were maintained in Dulbecco’s modified Eagle medium (DMEM) supplemented with 10% fetal bovine serum (DMEM; Gibco Thermo Fisher, Waltham, MA, USA, FBS; Hyclone, Logan, UT, USA), 2 mM L-glutamine (Gibco Thermo Fisher, Waltham, MA, USA), 1.5 g/L sodium bicarbonate (Gibco Thermo Fisher, Waltham, MA, USA), 100 U/mL penicillin (Gibco Thermo Fisher, Waltham, MA, USA) and incubated at 37 °C in 5% CO_2_. ZIKV strain PRVABC59 (ZIKV-PR; GenBank: KU501215) was originally isolated from a human traveler to Puerto Rico in 2015 and passaged three times on Vero cells prior to obtaining it from Aaron Brault (CDC, Ft. Collins, CO, USA). ZIKV strain DAK 41525 (GenBank: KU955591.1), passaged twice on Vero cells, was obtained from Greg Ebel (Colorado State University, Ft. Collins, CO, USA).

### 4.2. Ethics Statement and Animals

Use of all animals was approved by the Colorado State University Institutional Animal Care and Use Committee (protocol 16-6468A). All procedures were done in accordance with the Guide for the Care and Use of Laboratory Animals of the National Institutes of Health.

NZW rabbit and the Hartley guinea pigs were obtained from Charles River. The multimammate mice (*Mastomys natalensis*) were obtained from Heinz Feldmann (Chief, Laboratory of Virology NIH, NIAID, Rockville, MD, USA) provide from a breeding colony maintained by the Rocky Mountain Veterinary Branch, Division of Intramural Research National Institutes of Allergy and Infection Disease, National Institute of Health. A129 mice were also obtained from the Colorado State University breeding colony. As the first published study characterizing experimental infection of multimammate mice with BSL-3 viruses, it is worth noting that these animals are particularly fractious and must be handled with care. To this end, these animals were never handled unless fully anesthetized.

### 4.3. ZIKV Subcutaneously Inoculation into Animals

Four A129 mice and 12 multimammate mice, 8–12 weeks old, were anesthetized in a holding chamber with 1–3% isoflurane to effect with an oxygen flow rate of 1.5 L/min. Once the animal was anesthetized, it was removed from the chamber and 2.6 × 10^6^ PFU (100 µL) ZIKV or PBS (100 µL) was administered subcutaneously between the scapulae with a sterile hypodermic 34-gauge needle in a biosafety cabinet. As the highest dose available to us, 2.6 × 10^6^ PFU was selected as an inoculation dose to ensure that if these animals were suspectable to ZIKV an infection would be detected. Sexually mature 6-month-old male rabbits were restrained by one researcher while the other used a sterile hypodermic 34-gauge needle to subcutaneously inoculate 2.6 × 10^6^ PFU (100 µL) of virus or PBS (100 µL) between the scapulae. Four sexually mature male guinea pigs 8–12 weeks of age were inoculated subcutaneously with 2.6 × 10^6^ PFU (100 µL) of ZIKV PRVABC59 or PBS (100 µL). Animals were restrained by one researcher while the other used a sterile hypodermic 34-gauge needle to subcutaneously inoculate 2.6 × 10^6^ PFU (100 µL) of virus between the scapulae. As these animals were previously shown to be susceptible, another four male guinea pigs were inoculated by infectious mosquito bite (one was fed on by non-infectious mosquitoes) to evaluate if mosquito saliva would potentiate infection (see below for methods). All animals were individually housed to ensure that transmission did not occur between animals by another route.

### 4.4. Mosquito Infections of Guinea Pigs

To infect mice by mosquito bite, *Aedes aegypti* strain Poza Rica mosquitoes were fed an infectious blood meal and held for 14–17 days to ensure dissemination of virus to the salivary glands. Infectious blood meals were prepared with 1mL fresh virus contained in the cell culture supernatant of infected Vero cells and 1 mL of defibrinated calf blood. Back titration of the bloodmeals ranged between 1 × 10^6^–5 × 10^6^ PFU/mL. Mosquitoes were sorted post-feeding and 10–20 blood fed mosquitoes were placed in cartons with an organdy cover and provided water and sugar source. To allow the mosquitoes to feed on the guinea pigs, each guinea pig was anesthetized using 100 mg/kg ketamine combined with 10 mg/kg xylazine and placed on the organdy cover of one carton for ~20 min. After allowing the mosquitoes to feed on the guinea pigs, blood-fed mosquitoes were immediately knocked down, their saliva was collected by the forced salivation method described previously [60], and their bodies homogenized in media for later testing. ZIKV infections of mosquito bodies were determined by plaque assay and qRT-PCR. Samples were titrated by Vero cell plaque assay, with a tragacanth gum overlay and staining at day 5 post-cell culture inoculation.

### 4.5. Intravaginal Inoculation of Female Rabbits

For intravaginal inoculation, two female rabbits (6-month of age) were restrained in a seated position by one researcher. Another researcher used a blunt 200 µL pipette tip to gently inoculate 2.25 × 10^4^ PFU (100 µL) ZIKV of virus into the vagina. Each rabbit was held in this seated position for 3 min to help facilitate absorption.

### 4.6. Sample Collection: Urine, Rectal Swab, Oral Swabs, Semen, and Blood

A129 mice and multimammate mice: 20–50 µL of blood was collected every two days from all mice by a small nick in the lateral tail vain. A129 Mice were restrained in a mouse restrainer during bleeding and due to fractious nature of multimammate mouse, only blood and saliva samples were taken when the mice were anesthetized no other ante-mortem samples were collected

NZW Rabbits: Urine, rectal swabs, oral swabs, and blood were collected every two days. Urine was collected as produced during handling, animals were manipulated over plastic wrap, and if urine was released it was pipetted off the plastic wrap and placed into 1.7 mL tube and put directly onto dry ice. Rectal swabs and oral swabs were taken while animals were restrained by one researcher. Blood was collected from a venipuncture performed on the marginal ear vein. Semen was collected from male rabbits every two days. Semen was collected from rabbits only using an artificial vagina made from 2 inches of PVC pipe. PVC was lined with a plastic tube seal on the outside of one end. The plastic liner was filled with hot water and the other side was sealed. In one end, a 15 mL conical tube was placed the other end was lubricated. A male rabbit was placed into the cage with the female and once interest was expressed by the male, the researcher placed the artificial vagina between the male and female. The rabbit’s penis was guided into the artificial vagina and semen was collected upon ejaculation.

Guinea pigs: Urine, rectal swabs, oral swabs, and blood were collected every two days. Urine was collected as produced during handling. Animals were manipulated over plastic wrap, and if urine was released it was pipetted off the plastic wrap and placed into 1.7 mL tube and put directly onto dry ice. Rectal swabs and oral swabs were taken while animals were restrained by one researcher. Blood was collected by cranial vena cava venipuncture.

### 4.7. Body Weight and Temperature

Body weight was taken daily first thing in the morning (q24 h) for rabbits and guinea pigs. Animals were placed on a scale and weight was recorded. Rectal temperatures for rabbits and guinea pigs were taken with a lubricated standard thermometer.

### 4.8. Euthanasia, Blood Collection and Necropsy

A129 mice and multimammate mice were euthanized by cervical dislocation following inhalation anesthesia using isoflurane. Cardiac blood was collected with a 34-gauge sterile needle inserted into the apex of the heart.

The NZW rabbits and guinea pigs were euthanized by overdose of ketamine and xylene consistent with Institutional Animal Care and Use Committee recommendations.

For all animals, pieces of each tissue were removed and placed in a pre-weighed tube with 500 µL of DMEM media and kept at 80 °C for RNA extraction. Tissue for each animal included saliva, blood, and tissues including brain, heart, lungs, liver, kidney, bladder and reproductive tract (ovary, testis, and seminal vesicles for rodents).

### 4.9. RNA Extractions

Tubes containing pieces of tissue were re-weighed, homogenized for 1 min at 24 c/s, and spun for 5 min at 14,000× *g*. RNA was extracted from all samples using the Mag-Bind Viral DNA/RNA 96 kit (Omega Bio-Tek, Norcross, GA, USA) on the KingFisher Flex Magnetic Particle Processor (Thermo Fisher Scientific, Waltham, MA, USA). RNA was eluted in 30  µL nuclease-free water.

### 4.10. qRT-PCR

Promega GoTaq Probe 1-Step RT-qPCR System was used on RNA extracted from blood and tissues to quantify ZIKV RNA according to manufacturers’ instructions. Primers targeting the NS5 gene were used (ZIKV 1086 (CCGCTGCCCAACACAAG) and ZIKV 1162c (CCACTAACGTTCTTTTGCAGACAT)). The probe used was ZIKV 1107-FAM (AGCCTACCTTGACAAGCAGTCAGACACTCAA) [61]. Standards for ZIKV were generated by establishment of PFU equivalence. RNA was extracted from stock virus with a known viral titer and was diluted to achieve serial 10-fold PFU equivalence dilutions. The standard curve of 10^3^–10^8^ ZIKV PFU equivalence/reaction and had a primer efficiency of 88.62% with an R^2^ value of 0.971, a slope of −3.629, and y-intercept = 47.270.

### 4.11. Plaque Reduction Neutralization Test (PRNTs)

PRNTS were performed on serum that was heat-inactivated by incubating at 56 °C for 30 min. Samples were serially diluted (ten-fold) into DMEM media and mixed with ZIKV strain PRVABC59. Serum and virus were incubated at 37 °C for one hour and then plated onto confluent Vero cells and incubated for one more hour at 37 °C. Tragacanth gum overlay was added, and cells were stained at 5 days post-inoculation.

### 4.12. Statistical Analyses

Results in figures express individual values. The statistical details are noted in the figures and/or in the corresponding figure legends. Multiple t-test was used to compare inoculated animals to control animal/s at each time point in the GraphPad Prism (GraphPad Software, La Jolla, CA, USA).

## Figures and Tables

**Table 1:** Summary of all animal inoculations and sample collections.

Animal	n	Sex	Inoculation Route	Animals Per Inoculum Group			Euthanasia Timepoint	Samples Collected from Each Animal		
				ZIKV 41525	ZIKV PRVABC59	Sham-Inoculated		Ante-Mortem Samples **	Non-Reproductive Organs ***	Reproductive Organs
Multimammate mouse (*Mastomys natalensis*)	6	F	SC	2	3	1	5 dpi	Blood, Saliva	Brain, heart, lungs, liver, spleen, kidney, bladder	Ovary
	6	M		2	3	1				Testes, Seminal vesicles
New Zealand white rabbit (*Oryctolagus cuniculus*)	2	F	Ivag	0	2	0	Not euthanized	Blood, Saliva, Vaginal swab, Urine		N/A
	6	M	SC	0	4	2	7 dpi, 28 dpi *	Blood, Saliva, Semen Urine		Testes, Seminal vesicles
Hartley guinea pig (*Cavia porcellus*)	8	M	SC	0	3	1	7 dpi	Blood, Saliva, Urine		Testes, Cowper’s gland
			MB		3	1				

* for each timepoint, 2 inoculated rabbits and 1 sham-inoculated rabbit where euthanized, ** ante-mortem samples were collected every two days, *** no organs were collected from female rabbits, Ivag = intravaginal, SC = subcutaneous, MB = mosquito bite. Sham-inoculated animals (with 100 µL of PBS) were negative controls. N/A = non-applicable, ZIKV PRVABC59 = ZIKV strain PRVABC59 (ZIKV-PR; GenBank: KU501215), ZIKV 41525 = ZIKV strain DAK 41525 (GenBank: KU955591.1).

**Table 2:** Multimammate mice (*Mastomys natalensis*) inoculations.

Animal ID	Species	Sex	Virus Inoculated
1	*M. natalensis*	Male	Zika 41525
2	*M. natalensis*	Male	Zika 41525
3	*M. natalensis*	Male	Zika PRVACB59
4	*M. natalensis*	Male	Zika PRVACB59
5	*M. natalensis*	Male	Zika PRVACB59
6	*M. natalensis*	Male	Mock
7	*Mus musculus* (A129 strain)	Male	Zika PRVACB59
8	*Mus musculus* (A129 strain)	Male	Mock
9	*M. natalensis*	Female	Zika 41525
10	*M. natalensis*	Female	Zika 41525
11	*M. natalensis*	Female	Zika PRVACB59
12	*M. natalensis*	Female	Zika PRVACB59
13	*M. natalensis*	Female	Zika PRVACB59
14	*M. natalensis*	Female	Mock
15	*Mus musculus* (A129 strain)	Female	Zika PRVACB59
16	*Mus musculus* (A129 strain)	Female	Mock

All animals were subcutaneously inoculated with 2.6 × 10^6^ PFU in 100 µL of virus or 100 µL of PBS (Mock) and euthanized at 5 dpi. Mus musculus (A129 strain) were used as positive control. ZIKV PRVABC59 = ZIKV strain PRVABC59 (ZIKV-PR; GenBank: KU501215), ZIKV 41525 = ZIKV strain DAK 41525 (GenBank: KU955591.1).

**Table 3:** Study design for the inoculation of New Zealand white rabbits with ZIKV.

Animal ID	ZIKV Strain	Sex	Euthanized dpi
Rabbit 1	Zika PRVACB59	Male	7
Rabbit 2	Zika PRVACB59	Male	7
Rabbit 3	Mock	Male	7
Rabbit 4	Zika PRVACB59	Male	28
Rabbit 5	Zika PRVACB59	Male	28
Rabbit 6	Mock	Male	28
Rabbit 7	Zika PRVACB59	Female	N/A
Rabbit 8	Zika PRVACB59	Female	N/A

All animals were subcutaneously inoculated with 2.6 × 10^6^ PFU in 100 µL of virus or 100 µL of PBS (Mock). Females were not euthanized after 28 days; saliva, urine and vaginal swabs were negative by qRT-PCR (N/A = non-applicable). ZIKV PRVABC59 = ZIKV strain PRVABC59 (ZIKV-PR; GenBank: KU501215).

**Table 4:** Inoculations of ZIKV into adult male guinea pigs (GP).

Animal ID	ZIKV Strain	Inoculation Route	Sex
Guinea Pig 1	Zika PRVACB59	SC	Male
Guinea Pig 2	Zika PRVACB59	SC	Male
Guinea Pig 3	Zika PRVACB59	SC	Male
Guinea Pig 4	Mock	SC	Male
Guinea Pig 5	Zika PRVACB59	MB	Male
Guinea Pig 6	Zika PRVACB59	MB	Male
Guinea Pig 7	Zika PRVACB59	MB	Male
Guinea Pig 8	Mock	MB	Male

All animals were euthanized at 7 dpi. SC = subcutaneous, MB = mosquito bite. SC inoculated animals were subcutaneously inoculated with 2.6 × 10^6^ PFU in 100 µL of virus. Mock animals were negative controls and inoculated with 100 µL of PBS or fed on by non-infectious mosquitoes.
